# The Barrier to Accessing Dental Healthcare Services Among the Institutionalized Visually Impaired Adults: A Qualitative Study

**DOI:** 10.7759/cureus.60719

**Published:** 2024-05-20

**Authors:** Samikshya Jena, Gunjan Kumar, Ranjanmani Tripathi, Sourabh Khandelwal, Oshin Sharma, Shivani Arora

**Affiliations:** 1 Department of Public Health Dentistry, Sai Laparoscopic Hospital, Rourkela, IND; 2 Department of Public Health Dentistry, Kalinga Institute of Dental Sciences, Bhubaneswar, IND; 3 Public Health Dentistry, Index Institute of Dental Sciences, Indore, IND; 4 Prosthodontics and Crown & Bridge, Index Institute of Dental Sciences, Indore, IND; 5 Department of Orthodontics and Dentofacial Orthopedics, Government Autonomous College of Dentistry, Indore, IND; 6 Department of Prosthodontics and Crown & Bridge, Desh Bhagat Dental College and Hospital, Mandi Gobindgarh, IND

**Keywords:** healthcare service, visually impaired, dental professional, oral health, barrier

## Abstract

Many studies have focused on the overall oral health of people with visual impairment, but there is a dearth of studies on the barriers to accessing dental healthcare services among institutionalized visually impaired people. Therefore, the current study aims to assess the barriers to accessing dental healthcare services among institutionalized visually impaired people.

Methods: A qualitative study design was conducted over the course of 10 months among institutionalized visually impaired individuals. A semi-structured interview was conducted among the participants. Interviews were audio-recorded, transcribed, translated, and qualitatively analyzed using MAXQDA software, version 22.0 (VERBI Software, Berlin).

Results: A total of 20 participants participated in the study. Three levels were used to classify the investigated barriers: the individual's level, which pertains to the obstacles they encountered in receiving oral health care and their viewpoints on the way that care is provided; the interpersonal degree and the system level, in order to determine the broader components and their impact.

Conclusion: This study gives insight into the problems people have in assessing the dental services and facilities available. Three levels were used to identify the barriers among the study participants. Six themes emerged in the study that described their problems, which affected their mental health directly.

## Introduction

Oral health is an essential measure of general health, well-being, and quality of life. It covers a wide range of oral health-related diseases and conditions. According to the 2019 Global Disease Burden Survey, around 3.5 billion people worldwide are thought to be impacted by oral disease [1].

Vision impairment, as defined by the National Institutes of Health (NIH), is defined as a chronic impairment of vision that interferes with everyday activities and is not treatable with conventional eyeglasses or contact lenses. It is a visual issue that can cause reduced eyesight or even total blindness [2]. Almost 2.2 billion people worldwide suffer from some form of visual impairment; of those, at least 1 billion have an illness that could have been avoided or is still untreated [3]. The most important factors contributing to vision impairment and blindness are cataracts and uncorrected refractive errors. Though vision loss can affect people of different ages, predominantly people over 50 years of age experience vision impairment and blindness. To combat this issue, numerous worldwide initiatives have been developed [4]. By implementing several beneficial steps under its continuing National Programme for Control of Blindness and Visual Impairment, India has dramatically decreased the incidence of vision issues in recent years [5]. An important issue that must be taken into account while working with visually impaired people is compliance, according to a health affairs article. These obstacles often take the form of limitations on orientation as well as unavailable interaction or data [6].

Patients who are visually impaired represent a special group that presents challenges to the dentist's abilities and expertise. Oral health may be impacted by visual impairment due to physical, psychosocial, or cognitive challenges associated with the impairment, accompanying medical problems, or an absence of knowledge in a manner that is appropriate. Due to the difficulties in maintaining excellent dental hygiene, those with visual impairments are more likely to develop oral disorders, specifically periodontal disease. Dental issues are only apparent once they begin to feel discomfort or any other difficulty [7].

Significant obstacles prevent people with vision loss from getting the care they need, which leads to established health disparities. The lack of physical accessibility is one of the major obstacles. A dearth of services, inadequate financing or resources, inadequate transportation, a lack of social awareness, or inadequately educated or trained service providers are among other challenges [8]. Individuals in their immediate vicinity, such as relatives and companions, play a crucial role in ensuring that they receive the dental services they need because they need company to get there. So, it is not just the individual's willingness but also the ability of others around them to give them the opportunity to do so [9]. To increase access to dental services, measures can be taken, such as maintaining clear pathways, making sure spaces are well-lit, clearly defining door frames and handles, and installing handrails near staircases or lifts. Maintaining clear pathways, making sure areas are well illuminated, clearly defining door frames and knobs, and installing handrails by escalators or elevators can all be done to promote access to dental services.

The focus of qualitative research is on understanding a research question from a humanistic or idealistic perspective. Understanding people's views, perceptions, opinions, behavior, and connections involves using the qualitative technique. It produces non-numerical data. The incorporation of qualitative research into intervention studies is receiving increased attention from researchers across multiple disciplines.

To our knowledge, there have been no studies conducted on the barriers to accessing dental healthcare services among institutionalized visually impaired people. Given the above, this current study aims to assess the barriers to accessing dental healthcare services among institutionalized visually impaired people.

## Materials and methods

Study design, study population and inclusion criteria

A qualitative study was carried out among the visually impaired individuals at the Orissa Association of Blind Bhubaneswar City, Odisha. The principle of maximum diversity was maintained to select the participants. Patients between the ages of 18 years and above, those who were present at the time of the study, and individuals who gave informed consent were included in the survey. Subjects who refused to give consent were not involved in the survey.

Ethical approval and consent

The study was granted approval by the Institutional Ethics Committee (IEC) at Kalinga Institute of Medical Sciences Bhubaneswar, Odisha, under reference number KIIT/KIMS/IEC/1306/2023. The purpose of the study was explained in detail to the participants, who consented verbally to participate and allowed audio recording of their interviews. The interviewees gave their verbal approval to participate and to have their interviews recorded on audio. The data were secured in an encrypted password device that was only accessible to the research team members.

Interviews

The interviews were carried out over the course of 10 months. Interviews lasted for 30 to 50 minutes and were conducted in the local language (Odia). An interview guide was prepared for the qualitative assessment by members of the research team based on the prevailing data and expertise of the team in this field. A semi-structured questionnaire was constructed based on the following topics: sociodemographic details and health perceptions regarding general and oral health. A panel of four professionals, including two dentists, a medical professional, and a biostatistician assessed the content validity. The reliability coefficient (Cronbach’s alpha) was found to be 0.813. For every single question, study participants were asked open-ended questions followed by subsequent probing questions to allow them to elaborate on the matter. Interview questions regarding the barriers to accessing dental healthcare services are presented in Appendix 1.

After obtaining proper consent from the participant, the interview was audio recorded through an audio recording device. The audio recording was conducted by two members of the study team who were trained and were aware of the objectives of the study. The participants were randomly divided among themselves so that each one got ample time to conduct the full interview without any disturbance and fatigue. The participants talked freely and elaborately about the barriers to assessing dental and general healthcare services in the local setting. Additionally, the interview began with the sociodemographic data of the participants, which consisted of age, level of education, marital status, occurrence of visual impairment (according to age), and degree of visual impairment.

Data analysis

First, a verbatim transcription of the audio recordings was performed. English translations of the audio were made. Two bilingual members of the research team went over each interview's text to make sure it matched the audio recording exactly. If there were any differences, the team discussed and resolved them. To protect their privacy, a code number was assigned to each participant. The data was entered into a Microsoft Excel spreadsheet, and any missing numbers were subsequently checked. MAXQDA software, version 22.0 (VERBI Software, Berlin) was employed in the analysis. The number of interviews was stopped once data saturation was reached, which was achieved after the 16th interview. To ensure that the sample contained the greatest amount of variation and to validate any apparent themes, four more interviews were conducted in addition to the data.

## Results

A descriptive data regarding the sociodemographic data of the study participants is provided in Table 1. A total of 20 participants participated in the study. The mean age of the participants was 37 ± 16.79, the prevalence of females (55.31%) was greater than males (44.69%), majority (77.86%) of the study participants had higher education level. Most of the participants were single (51.87%), more than half of the study participants had visual impairment since birth (61.73%) with complete blindness (73.07%).

**Table 1:** Descriptive statistics to summarize the sample characteristics (N=20)

Variable	M (SD)	n (%)
Age	37 ± 16.79	
Gender		
Male		09 (44.69)
· Female		11 (55.31)
Educational level		
Low (≤ higher secondary school)		07 (22.14)
High (> higher secondary school)		13 (77.86)
Marital Status		
Single		11 (51.87)
Married		08 (44.23)
Divorced		00
Widow		01 (3.9)
Occurrence of visual impairment ( according to age)		
Since birth		12 (61.73)
>1 - 5 years of age		05 (25.11)
>5 years of age		03 (13.16)
Degree of visual impairment		
Partial blindness		04 (26.93)
Complete blindness		16 (73.07)

**Figure 1:**
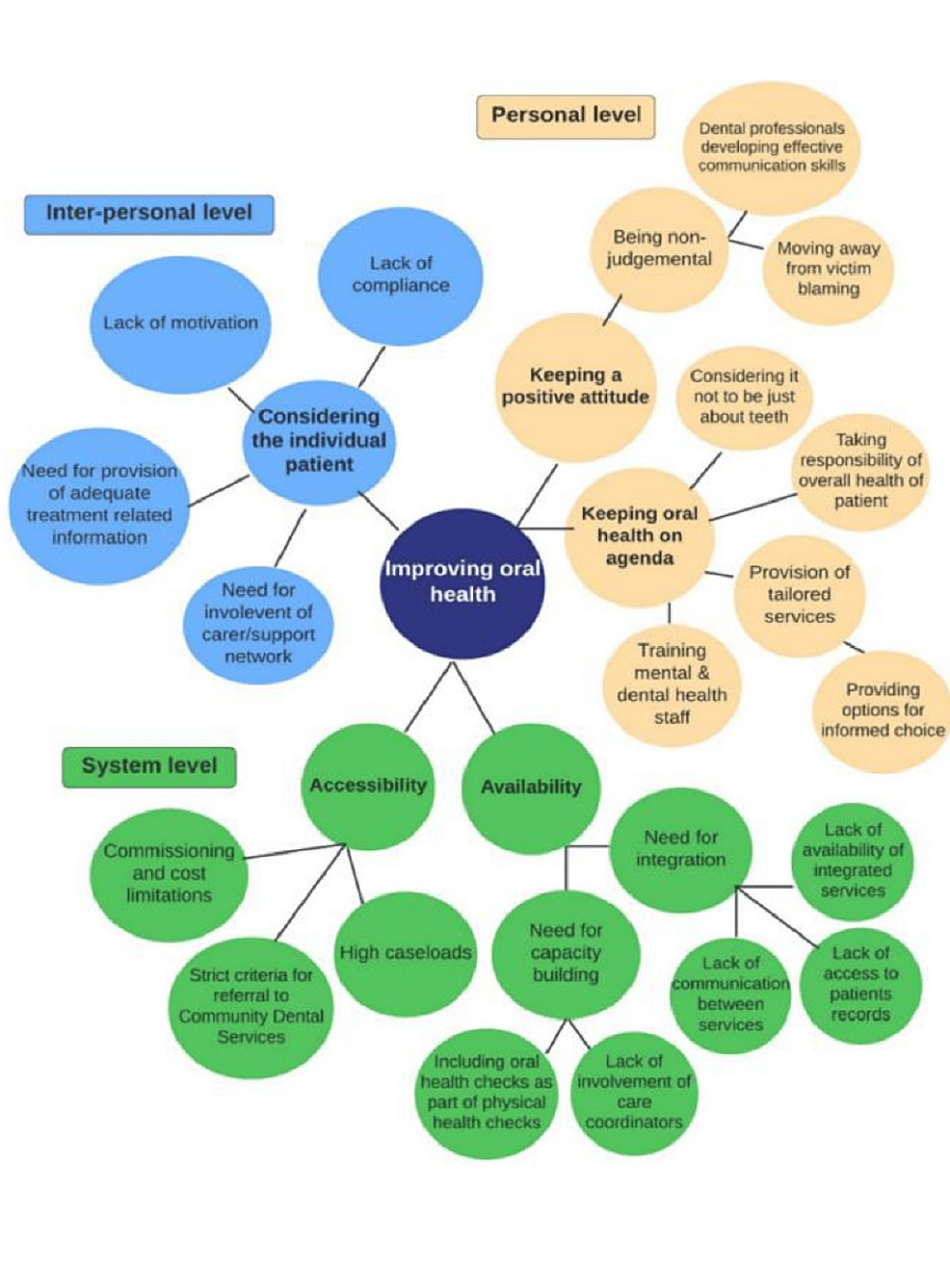
Thematic presentation of the investigated barriers Thematic presentation on the viewpoints on the obstacles to and suggestions for enhancing oral health in individuals at the individual, interpersonal, and system levels. The artwork is prepared by the authors.

Three levels were used to classify the investigated barriers: the individual's level, which pertains to the obstacles they encountered in receiving oral health care and their viewpoints on the way that care is provided; the interpersonal degree and the system level, in order to determine the broader components and their impact. As seen in Figure 1, the aggregated themes were arranged as sub-themes under each of the three levels. Analysis of the data revealed themes with sub-themes, and for this reason, thematic analysis findings are presented in combination (Table 2).

**Table 2:** Themes and Subthemes

THEME	SUB-THEME
Optimizing the situation	personal obstacles
Impact on oral health	high risk to oral diseases, intrusive aspect of dental treatments, spectrum of feelings while visiting a dentist
Positivity in Mindset	act of empathy, compassion, kindness, and understanding by the service provider; enhancement of communication skills
Maintaining the priority of oral health	adopting a holistic approach, training dental workers in mental health
Availability of all-inclusive assistance	all-encompassing assistance
Utilization of Dental Services	difficulties related to accessibility and the absence of integrated care

Theme 1: Optimizing the Situation

Sub-themes within this theme pertained to the personal obstacles that both service providers and clients experienced in relation to oral health.

Theme 2: Impact on Oral Health

Participants discussed how they were at higher risk when compared to the entire society. One instance of this was how the users' inability to sustain proper dental hygiene was impacted by their lack of drive. “I mean, I can spend days when I can’t actually get out of bed, never mind think about cleaning my teeth; you know that’s just not something that’s going to happen.” Additionally, because of linkages with unpleasant feelings or past experiences, the intrusive aspect of dental treatments was also mentioned as a significant barrier.

“I think it’s really a common thing like a lot of people have had experiences that you know felt very intrusive and as an invasive and around the mouth, it makes sense to me that, like dentistry is really triggering for that and really replicate some of that feeling of powerlessness feeling of being out of control, it being painful like having to have your mouth open and you’re not in control of that.”

The participants also discussed the spectrum of feelings they experience, including embarrassment, fear, worry, and anguish about dental procedures, whenever they had to see a dentist. It was emphasized that hearing the sounds of drilling while seated in the waiting area can cause uneasiness. From the interviews, it was clear that oral health was seen as a crucial component of overall health and well-being. The main obstacle that the service providers personally encountered was a lack of knowledge about their mental illness and a disregard for the need to treat each patient as an individual.

“This level of education is really needed with these groups of individuals around trauma, and you know, so that they are psychologically informed and trauma-informed. You know, who wants to put anybody through any kind of distress, but you know so it’s a group of people that really do need to learn more about their patient”.

Theme 3: Positivity in Mindset

The service providers also shared the opinions of the service users regarding the importance of subtlety and delicacy when interacting with patients who are visually impaired. They both felt that when working with patients, it was important to put judgment aside and act with empathy and compassion. They concurred that when interacting with patients, one must give up passing judgment and act with kindness and understanding.

In my opinion, it's crucial to avoid passing judgment on patients. Even if you could assume that a certain number of patients brush once or twice a week, it's truly not usual for them, and that's all I can ask of them. Thus, I believe it comes down to starting with the fundamentals and being practical and impartial.

The health experts also emphasized that one crucial area that needed enhancement was the acquisition of excellent communication skills in order to be able to speak with patients who needed additional assistance.

“So just as much as tooth brushing is a habit, it’s a healthy habit, and it needs to be encouraged, so again, it just comes back to the way in which that conversation happens. It’s not the ‘you need to do it like this’, we need ‘we’re here to educate you and tell you what to do’, it’s more ‘do you understand the benefits of what I am teaching you and can you demonstrate it to me so that I know that you’re able to do it well yourself’ and that’s the approach that I think could go somewhere.”

Theme 4: Maintaining the Priority of Oral Health

The necessity of proficient communication skills for patient management was emphasized by healthcare professionals, and this was investigated further to determine how it could be applied to the needs of dental health. It was advised that the best course of action would be to adopt a more holistic approach that would take into account the patient's requirements as an individual and not simply their teeth.

“I think that sometimes people may misunderstand that oral health just means mouth and teeth but actually it’s about the whole of the person, including medical but also including and I suppose it’s sort of taking a rounded approach to the person and sort of a holistic approach for that person.”

“I think education is quite the key and also trying to break down those barriers and say you know we are kind of patient people, we do understand your problems and anxieties and try to find ways of managing that and dealing with that and showing them that it’s not as bad as what they think is.”

Another topic that was brought up as needing attention to address the obstacles to giving patients the best care possible was the training of dental workers in mental health.

“Yes, indeed clinicians don’t tend to raise things if they’re a bit anxious about whether they’re able to deal with what comes up. So, I think there is a need for some mental health training for the dentist. Maybe even a two-day course in mental health first aid. Not expecting the dentists to train as mental health professionals, that’s a little bit of training we have.”

Theme 5: Availability of All-Inclusive Assistance

At an institutional level, it was evident that people want more all-encompassing assistance in order to get the treatment they need for their dental health.

Theme 6: Utilization of Dental Services

The service users highlighted two primary barriers at the service level: difficulties related to accessibility and the absence of integrated care. The patient mentioned how challenging it was to locate a dentist. Even if they were able to locate one, the dental office would either be too far away, creating problems with transportation, or they would be kicked out of the practice because of their condition-related missed appointments.

Another major obstacle to receiving dental care was stated to be the expense of the procedures. The missing component was identified as the lack of integration amongst health services with relation to providing holistic care and taking the patient's general health and well-being into consideration. The dental healthcare provider stated that because of this lack of integration, other health professionals did not prioritize oral health and did not take the detrimental effects of poor oral health into account.

It was advised to schedule a dental appointment quickly and conveniently, yet these services are currently in low supply. “The majority of people who come in, it isn’t that it was their focus or their priority, but if there were any issues there used to be a facility for a very quick referral to a local dentist and the whole system is not there anymore.” Health experts concurred with users that there was a lack of integration between services, with each provider primarily addressing a single area of patients' health rather than cooperating to enhance the patient's general health and well-being.

## Discussion

This survey shows that the special-needs population shares the most often mentioned hurdles (expense, fear, and lack of perceived need) and that fear/anxiety is a major barrier to the utilization of dental services among this demographic [9]. The following literature supports this finding: unusual dental appointments, explained as less than two visits in a year, were most frequently attributed to three factors, according to a US general population survey: value (38%), a deficit of recognized necessity (27%), and panic (17%) [10]. According to a Canadian population survey, there is a connection between having a greater degree of dental anxiety and not having seen the dentist in the previous five years, as well as between not having a regular dentist and having a history of skipping dentist appointments [11]. According to a survey done in a large US city, 15% of participants said they avoided going to the dentist for at least a year and had some degree of dental phobia [12].

The statistics also corroborate prior research showing a negative correlation between dental utilization and panic/apprehension [13], and a positive correlation between higher percentage of dental visits and well-rated oral health. As many special-needs patients reported that they would use oral health care services more frequently if anaesthesia and sedation services were available to them. This perception directly affects the frequency of dental visits [14].

Brushing your teeth is one of the most important dental hygiene practices that you should follow to keep your mouth healthy. It is advised that everyone clean their teeth twice a day, both throughout the day and before bed. The majority of the visually impaired participants in this study brushed their teeth once a day. Tooth decay can be effectively avoided by brushing with toothpaste containing fluoride. Consequently, tooth brushing should be done every day since it is helpful in the removal of plaque, which can cause gum disease [15]. Brushing teeth is not enough to keep the mouth healthy. Therefore, it is recommended to use interdental appliances in order to improve oral health. The American Dental Association recommends using floss to assist in cleaning in between teeth, which can aid in the prevention of gum disease and cavities. Approximately 54% of visually challenged people reportedly practiced dental hygiene with a toothbrush and toothpaste, according to a different study [16].

Thus, a more varied group of carers and healthcare professionals may be taken into account in future studies. The statistics also point to the necessity of conducting additional research on health care practitioners' attitudes, training, and resources with regard to providing dental treatment as well as outreach programme designed to assist marginalized members of society.

Limitations

The current qualitative studies had certain limitations. The sample size was small to represent a larger group of visually impaired people. Since this group was vulnerable, they were sceptical with their responses.

## Conclusions

This study gives an insight into the problems of the people to assess the dental services and facilities available. Three levels were used to identify the barriers among the study participants. Six themes emerged in the study that described their problems, which affected their mental health directly.

A thorough and systematic plan should be made to provide them with the best available dental care treatment by conducting camps and awareness programs, along with regular day-to-day check-up programs. Further models should be developed so that they are able to maintain proper oral hygiene and reduce the oral health problems with proper maintenance and care. Special facilities should be made for them to avail for the dental services avail near or around their localities. This qualitative study can help in further implementation of future policy and programme for the people with special need.

## Appendices

**Table 3:** Semi-structured interview guide This is the interview guide used to conduct semi-structured interviews. Open-ended questions (left-hand column) were used to initiate discussions while probing/closed-ended questions (right-hand column) were used to clarify or expand upon points made by participants based on the participant's response to the open-ended question.

Open-ended Questions	Follow-up/ Probing Questions
Do you think oral health is an important part of a healthy lifestyle.	Can you explain in detail what do you mean by (…)?
Did you receive dental treatment during last 12 months? Yes / no	If no- why didn’t they attend If yes- reason
Can you explain about the dental services available for your dental health?	Can you elaborate in detail?
Do you know about any dental Clinic or services available near to you?	If yes- mention some?
What do feel regarding problems for availing for oral health care facilities?	Have you experienced any such problem?
What can be done for better oral health services?	What are your suggestions regarding (…)?
